# Exposure to arousal-inducing sounds facilitates visual search

**DOI:** 10.1038/s41598-017-09975-8

**Published:** 2017-09-04

**Authors:** Erkin Asutay, Daniel Västfjäll

**Affiliations:** 1 Department of Behavioral Sciences and Learning, Linköping University, Linköping, SE-58183 Sweden; 2 Decision Research, Eugene, OR 97401 USA

## Abstract

Exposure to affective stimuli could enhance perception and facilitate attention via increasing alertness, vigilance, and by decreasing attentional thresholds. However, evidence on the impact of affective sounds on perception and attention is scant. Here, a novel aspect of affective facilitation of attention is studied: whether arousal induced by task-irrelevant auditory stimuli could modulate attention in a visual search. In two experiments, participants performed a visual search task with and without auditory-cues that preceded the search. Participants were faster in locating high-salient targets compared to low-salient targets. Critically, search times and search slopes decreased with increasing auditory-induced arousal while searching for low-salient targets. Taken together, these findings suggest that arousal induced by sounds can facilitate attention in a subsequent visual search. This novel finding provides support for the alerting function of the auditory system by showing an auditory-phasic alerting effect in visual attention. The results also indicate that stimulus arousal modulates the alerting effect. Attention and perception are our everyday tools to navigate our surrounding world and the current findings showing that affective sounds could influence visual attention provide evidence that we make use of affective information during perceptual processing.

## Introduction

Increased arousal can lead to increased vigilance that is reflected by the modulation of neural excitability of sensory cortices, which in turn facilitates attention^1, 2^. Hence, activity in the brain regions that are involved in active information processing can be regulated by the presence of affectively salient stimuli (e.g. ref. 3). It has earlier been found that exposure to an affective visual stimulus both enhances contrast sensitivity for subsequent visual stimuli^4^ and facilitates faster search for a subsequent neutral visual target^5^. In addition, induced arousal leads to a greater bandwidth of the visual contrast sensitivity function^6^. In the auditory domain, the presence of an emotionally arousing auditory-scene that is composed of concurrent environmental sounds leads to an increase in perceptual sensitivity in an auditory change-detection task^7^. We argue that these findings suggest that arousal induced by auditory and visual stimuli increases vigilance and alertness, which in turn leads to a decrease in attentional thresholds in respective perceptual modalities. Here, we studied a novel aspect of affective enhancement of attention – we tested whether auditory-induced arousal by task-irrelevant sounds could modulate visual attention in a subsequent search task.

Vigilance and alertness could be defined as the ability to achieve and maintain a state of high sensitivity to incoming stimuli. A common approach to study alertness is to employ a non-specific warning cue prior to a target that requires a speeded response. If the cue is effective in modulating phasic alertness, then reaction times improve^8, 9^. The alerting cue is usually non-spatial, so it only provides information regarding when a target might appear^10^. Previous research found that auditory phasic alerting could ameliorate spatial imbalance in visual attentional deployment in spatial neglect patients^11^. Studies on healthy participants found that auditory phasic alerting could improve conscious visual perception^12^ and increase attentional bias for salient visual stimuli^13^. In these studies, however, the alerting cues (tone bursts with various durations) have no motivational or affective value. In the present study, we employed environmental sounds as auditory alerting cues and investigated whether arousal induced by an auditory stimulus could modulate visual attention in a search task.

A related line of relevant research concerns crossmodal interaction studies that focus on the influence of affective or motivational value of a sound on visual perception. Auditory stimuli with high-reward associations can increase visual sensitivity in an orientation discrimination task compared to sounds with low-reward associations, which points to a value-driven crossmodal interaction that influences visual perception^14^. An increase in visual sensitivity during an orientation discrimination task was also induced by looming compared to receding sounds^15^. Looming sounds can induce biases in behavior and neural activity^16^, and are affectively more salient than receding sounds^17^. However, these reported crossmodal value- and saliency-driven effects on visual perception do not seem to stem from a change in vigilance or alertness, since auditory (i.e. the cue) and visual (i.e. the target) stimuli in these studies were presented simultaneously. Further, it was found that looming sounds has the capacity to increase phasic alertness for a visual stimulus using a version of the cue-target task^18^. However, in the light of a previous experiment that found an opposite effect^19^, the authors suggested that the phasic alertness effect might depend on central vision or audio-visual integration. In these studies, participants responded to a single visual or auditory stimulus following an auditory-cue (looming or receding stimuli) by pressing a button.

Earlier studies suggested that exposure to moderate to high levels of noise (higher than 80dBA) can facilitate selective information processing^20^. For instance, when exposed to noise during a central tracking task, performance increased for high probability targets while it decreased for low probability targets^21^. Further, in a combined tracking and multi-source monitoring task exposure to high noise led to decreased detection rates of peripheral signals^22^. These results have been interpreted as increased selectivity of attention with arousal induced by noise^20, 23^. In these studies, similar to auditory-phasic alertness studies, the auditory signals were noise. In addition, performance while exposed to noise was usually compared to a baseline with no auditory stimulus present. Moreover, these earlier results are not based on active visual search; thus, it is not entirely clear how search time, efficiency, and accuracy would be affected by auditory-induced arousal. In the current studies, we are interested in whether a facilitation of attention and increased search efficiency could be found when participants are exposed to environmental sounds, and whether the size of this effect depends on induced arousal levels by the auditory stimuli. We hypothesized that arousal value of the auditory-cue is the determining factor in attentional facilitation during visual search.

The affective significance of an object for an individual is related to its capacity to influence changes in one’s heartbeat, breathing, etc.^24^. An object or an event that is potentially important for our survival, relevant to our immediate goals, or salient and meaningful has a stronger ability to influence our body and mind state^25^. Much of the time the sensations that arise from the body are experienced as consciously available feelings of pleasure or displeasure associated with a certain degree of arousal; yet at other times, the elicited sensations can be very subtle and less available to consciousness^24, 25^. It has been suggested that hedonic valence (pleasure-displeasure) and arousal form a unified affective state (i.e. core affect; refs 25 and 26); and it can be subjectively described and represented as feelings of pleasure (or displeasure) and arousal. In the current study, we employed subjective measures to assess participants’ affective states in response to auditory cues. Participants were asked to report their momentary affective states (*“How do you feel right now?”*) on visual analog scales of valence and arousal after listening to each auditory cue (see Methods for details). We expected that the auditory cues inducing higher arousal would have a greater capacity to facilitate attention. This prediction is in line with previous research showing that exposure to affective stimuli and increased arousal could bias attention and perception^4–7, 27^.

**Figure 1:**
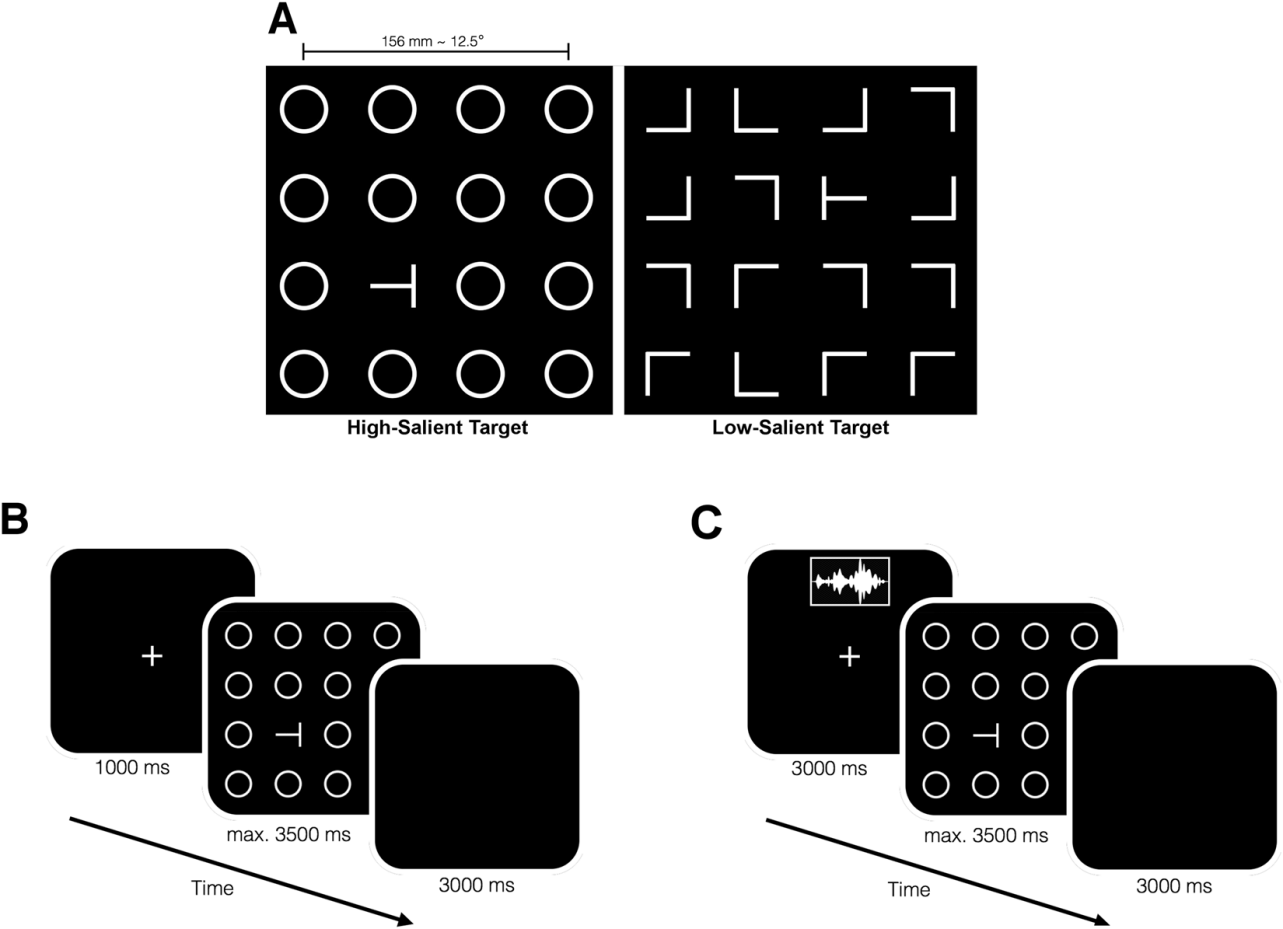
Visual search task details for Experiment 1. (**A**) The figure shows sample visual arrays for high-salient (left) and low-salient (right) target conditions. (**B**) Timeline for the visual search task is illustrated. Participants were given maximum 3500 ms to find the target. (**C**) Timeline for the search task with the auditory cues is illustrated. Sounds were presented during the fixation period. The sound offset was aligned with the start of the search period.

The auditory system is involved in scanning the surroundings, detecting salient events, and orienting the visual system. Based on these functions of the auditory system, and on the evidence suggesting that increased arousal can facilitate attention (e.g. ref. 7), we hypothesize that exposure to arousing sounds could facilitate subsequent visual processing and attention. In order to test our hypothesis, we adapted a visual search task, wherein participants were exposed to task-irrelevant environmental sounds prior to the search task. Participants actively searched for a symbol resembling a letter-T on its either side and indicated its orientation. We implemented two target saliency conditions (i.e. high-salient vs. low-salient) by using different distractors in the visual scenes. In the high-salient condition (HS), the distractors were circles, whereas they were Ls in different orientations in the low-salient (LS) condition (Fig. 1A). As a model for bottom-up attention, it has been suggested that several pre-attentive feature detection mechanisms process information in a parallel fashion over large portions of the visual field and encode spatial contrast for each feature. The feature maps, then, are combined into a salience map^28^, which can also be modulated by top-down factors such as relevance^29^. The salience map is scanned by deploying attention sequentially to locations of high contrast^28^. We expected that during HS, the target would stand out due to its high saliency, and the search times would not increase with increasing number of distractors. Whereas during LS, observers would need to actively deploy attentional resources over limited portions of the visual field to find the target among distractors, which would likely increase the search times as the number of distractors increase. Search slope, which is the average increase in search time for an added distractor, could reflect search efficiency^30^. In the first experiment, we investigated whether there is an effect of auditory-induced arousal on subsequent visual search and whether this effect is modulated by the spatial locations of the auditory-cue (front or rear) and the visual target (central or peripheral) and the target saliency (HS or LS). Whereas in the second experiment, we focused on search efficiency and investigated whether auditory-induced arousal can modulate search slopes.

## Results

### Experiment 1

In the first experiment, we used four different environmental sounds (see Methods for details) as task-irrelevant auditory-cues. Participants first listened to the auditory stimuli and were asked to report their momentary affective state after each sound using visual analog scales of valence (negative/positive affect) and arousal (high/low arousal). After the first session, participants performed two versions of the visual search task: the visual-only version (VO) and the search with task-irrelevant auditory cues (VA). In the VA version, one of the four auditory stimuli were presented during visual fixation; and a visual search array appeared on the screen after the sound (see Methods for details). Visual arrays contained one target and 15 distractors laid out on a 4 × 4 array (Fig. 1A). The experimental factors during the VO version were: target saliency (high-salient or low-salient) and target location (central or peripheral). Whereas, the factors in the VA version were: target saliency, target location, auditory cue, and sound source location (front or rear). The source location factor was introduced, since a previous experiment reported an auditory bias towards the rear perceptual field at both attentional and emotional levels^31^. Here, we aimed to study a possible sound location effect on visual attention.

#### Affective reactions to sounds

Affective reactions to auditory stimuli were scaled between −1 and +1, and entered into an ANOVA with stimulus (4 stimuli) and location (frontal vs. rear) within-subject factors (see Supplemental Table 1 for average ratings). Stimulus main effect was significant for both valence (F(3.84) = 18.56, p < 0.001, η_p_^2^ = 0.4) and arousal (F(3.84) = 15.4, p < 0.001, η_p_^2^ = 0.36). Post-hoc comparisons showed that on average fire-alarm and growling-dog were more negative and arousing compared to clucking-hen and microwave-oven (at p < 0.01 level). Location main effect and the interaction were non-significant.

#### Visual search

RTs during the VO version were analyzed using an ANOVA with target-saliency (HS or LS) and target-location (central or peripheral) within-subject factors. Both main effects (target-saliency: F(1.28) = 142.5, p < 0.001, η_p_^2^ = 0.84; target-location: F(1.28) = 63, p < 0.001, η_p_^2^ = 0.69) were significant, which indicated that participants were faster for HS (640 ± 40 ms) compared to LS (1114 ± 101 ms), and when locating central (784 ± 57 ms) compared to peripheral targets (970 ± 80 ms). The interaction effect was also significant (F(1.28) = 52.5, p < 0.001, η_p_^2^ = 0.65). When target location comparisons were carried out for the two target-saliency conditions separately, we found that target-location effect was present only during LS (F(1.28) = 64.5, p < 0.001, η_p_^2^ = 0.70). Since during LS the observers need to deploy attention onto limited portions of the visual array sequentially, it takes longer time to locate the target when it is placed further away from the central fixation. Whereas, during HS the distance from the central fixation did not affect the search times.

Finally, the analyses of accuracy data revealed that participants tended to be more accurate (F(1.28) = 3.93, p = 0.057, η_p_^2^ = 0.12) while locating central (0.985 ± 0.005) compared to peripheral targets (0.963 ± 0.009). Target saliency effect or the interaction did not reach significance for accuracy (p > 0.25).

#### Effect of auditory-induced arousal on visual search

**Figure 2:**
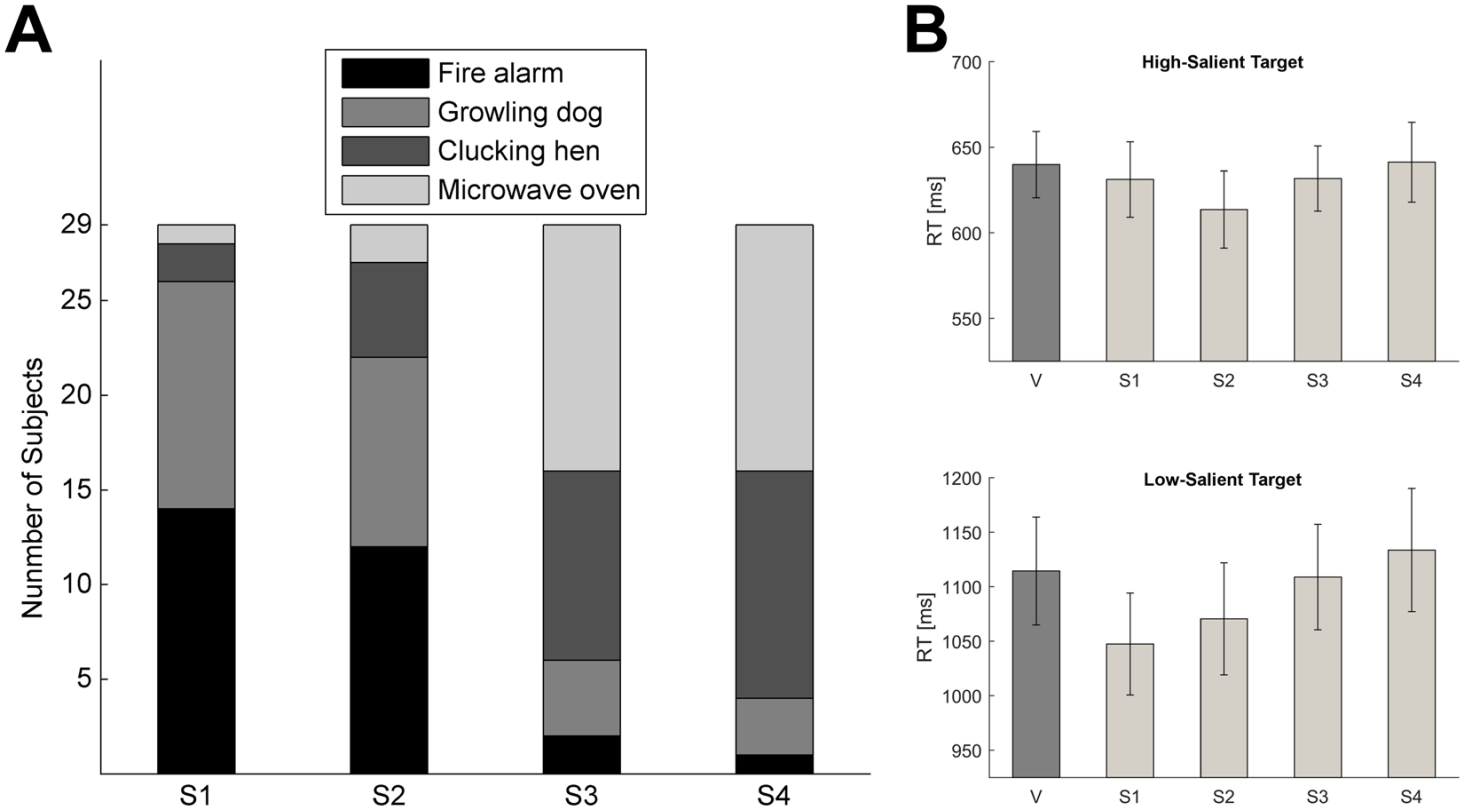
Results of Experiment 1. (**A**) The auditory stimuli were placed in order depending on the individual arousal ratings. The figure illustrates the stimuli in the order of decreasing arousal. S1 in the figure is the most arousing stimulus for each participant. (**B**) Average RTs during different target saliency conditions with (S1–S4) and without (V) the auditory stimuli (error bars represent standard errors).

To investigate the influence of auditory-induced arousal on RTs, we ordered the stimuli based on individual arousal ratings before submitting the RT data into an ANOVA (S1 to S4, see Fig. 2A and Methods for details). Hence, S1 is the most arousing stimulus for each participant. Similarly, S4 is the least arousing stimulus for each participant. RTs were analyzed using ANOVA with auditory-cue (S1 to S4), sound-location (frontal vs. rear), target-saliency, and target-location as within-subject factors. Here, we also looked for *a priori* significant linear contrasts of the auditory-cue main effect and its interactions to study the effect of auditory-induced arousal. Expected effects of target-saliency (F(1.28) = 172.6, p < 0.001, η_p_^2^ = 0.86), target-location (F(1.28) = 134.6, p < 0.001, η_p_^2^ = 0.83) and their interaction (F(1.28) = 146.8, p < 0.001, η_p_^2^ = 0.84) were found. Critically, auditory-cue main-effect (F(3.84) = 4.26, p = 0.008, η_p_^2^ = 0.13; Fig. 2B) and auditory-cue*target-saliency interaction (F(3.84) = 3.007, p = 0.035, η_p_^2^ = 0.097) were significant. Contrast analysis provided only a significant linear contrast of the auditory-cue main-effect (F(1.28) = 13.89, p = 0.001, η_p_^2^ = 0.33), indicating that RTs were reduced with increasing arousal value of the auditory-cue. Also, the linear contrast of the auditory-cue*target-saliency interaction was significant (F(1.28) = 10.12, p = 0.004, η_p_^2^ = 0.26), indicating that RT difference between the two target-saliency conditions was reduced with increasing auditory-induced arousal (see Table 1 for average RTs in each condition). None of the other main effects or interactions reached significance. To study the auditory-cue*target-saliency interaction further, we analyzed LS and HS separately. As a result, linear contrast of the auditory-cue main effect was significant for only LS (F(1.28) = 14.36, p = 0.001, η_p_^2^ = 0.34). Taken together, these findings indicated that search times reduced with increasing auditory-induced arousal while searching for a low-salient target but not for a high-salient target (Fig. 2B).

**Table 1:** Average reaction times for different target saliency conditions and auditory-cues in Experiment 1 in milliseconds.

	High-Salient Target		Low-Salient Target	
	RT [ms]	Accuracy [%]	RT [ms]	Accuracy [%]
Visual	640 (40)	97.8 (1.2)	1114 (101)	97 (1.5)
S1	631 (45)	99.1 (0.8)	1047 (96)	97.2 (2.2)
S2	615 (46)	98.3 (1.4)	1070 (105)	96.6 (2.4)
S3	632 (39)	98.1 (1.1)	1109 (99)	96.8 (1.8)
S4	641 (48)	97.2 (1.5)	1133 (116)	97.6 (2)

(95CIs are indicated in parentheses).

The analyses of the accuracy data revealed an almost significant linear contrast of the auditory-cue*target-saliency interaction (F(1.28) = 3.88, p = 0.059, η_p_^2^ = 0.12). The results showed that hit rates increased with increasing arousal rating of the auditory cue during HS condition but not in LS condition (Table 1). Whereas, with respect to RTs the effect of arousal was only found in the LS condition.

#### Valence effects

Since valence and arousal ratings for the auditory stimuli were negatively correlated (see Supplemental Table 1), we performed a control analysis to assess whether the findings presented above were largely due to stimulus arousal but not valence. We repeated all the statistical analyses after ordering the auditory stimuli for each participant according to their valence ratings. As a result, none of the main effects of or interactions with the auditory-cue factor reached significance (p > 0.15).

### Experiment 2

In Experiment 2, we introduced different set sizes to study the effect of auditory-induced arousal on search slopes. Furthermore, we removed target location and sound location factors that we used in the first study. The former was removed, since sound arousal did not seem to modulate search times depending on the target location. While the latter was removed, for there was no evidence for a sound source location effect in the first experiment. Another critical change was the increased number of auditory-cues in the second study. We used 24 different sounds in order to investigate whether the effect depends on certain physical features of the four sounds used in Experiment 1 or it replicates with other sounds as well.

**Figure 3:**
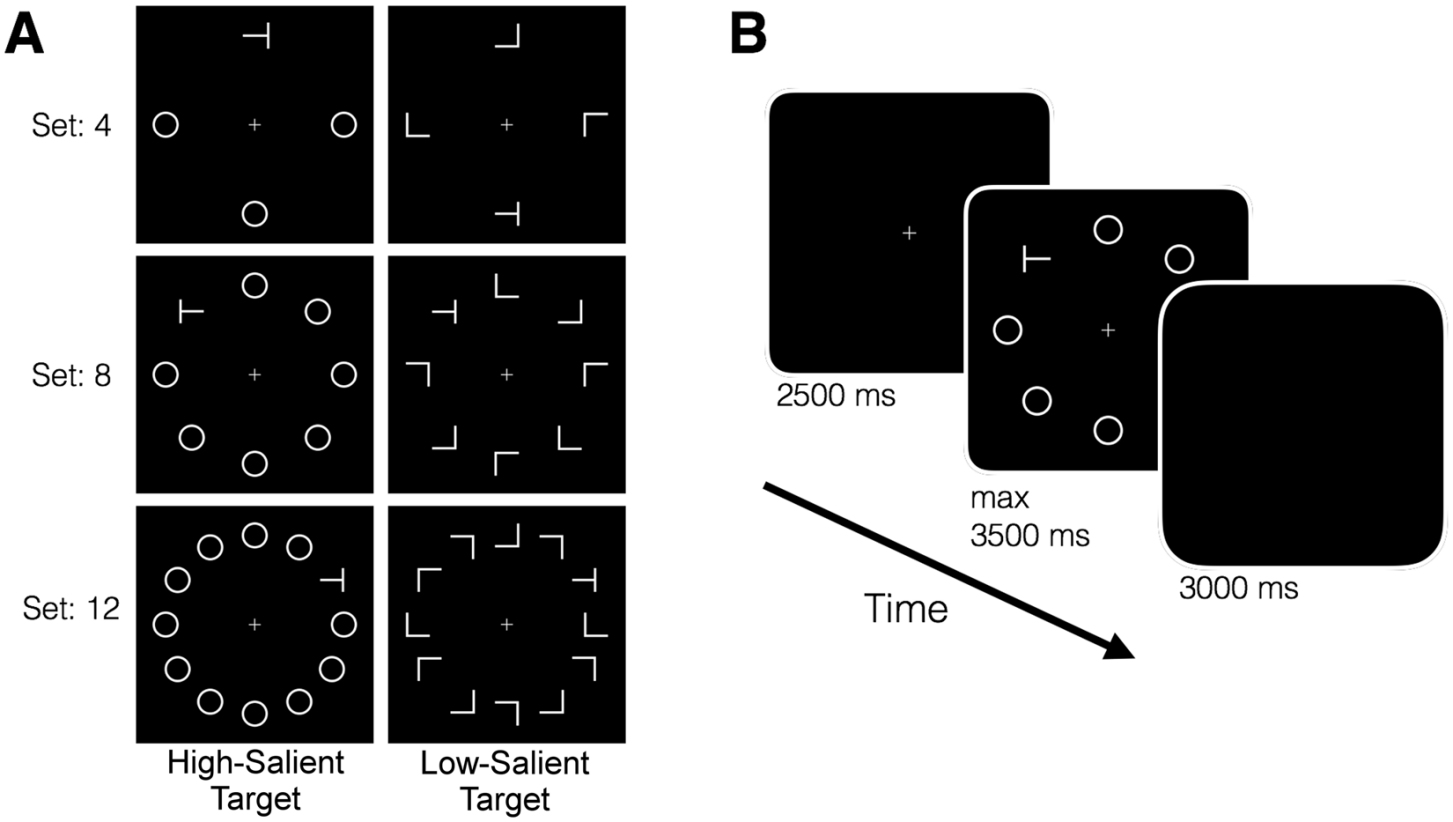
Task details of Experiment 2. (**A**) Sample search arrays for different set sizes and target saliency conditions. The center of each item was located 65 mm (ca. 5.3°) from the central fixation. (**B**) Timeline of the search task: Search arrays presented after a 2500-ms fixation. For the task version that included the auditory cues, a sound was presented during the fixation period, and the search array presented at the sound offset. For both versions participants were given a maximum of 3500 ms to find the target. Next trial started after a 3000-ms inter stimulus interval.

Similar to Experiment 1, participants first listened to the auditory stimuli and were asked to report how they felt after each sound using the same scales. Then, they performed VO and VA versions of the visual search task. In the VA version, an auditory stimulus was presented before the search array (see Methods for details). Items in search arrays were laid out on a circle at equal distance from the central fixation (Fig. 3A). The experimental factors for the search task were target saliency (high-salient or low-salient) and set size (4, 8 or 12). To investigate the influence of auditory-induced arousal on RTs, we ordered the stimuli based on individual arousal ratings. We, then, divided the 24 sounds into three different groups of eight stimuli for each individual (i.e. Low, Mid, and High arousal; see Supplemental Table 2 for mean valence and arousal ratings and respective error terms, one can also see the frequency of each stimulus classified in respective stimulus arousal categories). After forming these groups, we computed mean RTs for each set size, target saliency, and stimulus arousal. Finally, we calculated search slopes for each target saliency and stimulus arousal.

#### Visual Search

First, we analyzed data from the VO version. RTs were entered into an ANOVA with target saliency (HS or LS) and set size (4, 8, or 12) as within-subject factors. Both main effects (target saliency: F(1.25) = 327, p < 0.001, η_p_^2^ = 0.93; set size: F(2.50) = 134, p < 0.001, η_p_^2^ = 0.84) were highly significant. These effects indicated that participants were faster during HS (549 ± 29 ms) compared to LS (987 ± 59 ms), and that RTs increased with increasing set size (4-items: 642 ± 34 ms; 8-items: 762 ± 44 ms; 12-items: 901 ± 52 ms). The interaction effect was also significant (F(1.25) = 160, p < 0.001, η_p_^2^ = 0.87) indicating that the RT difference caused by different set sizes were larger in LS compared to HS. Moreover, search slopes were analyzed using an ANOVA with target saliency (HS or LS) and step (slopes between 4-to-8 or 8-to-12 items) as within-subject factors. Target saliency main effect was highly significant (F(1.25) = 239, p < 0.001, η_p_^2^ = 0.91), indicating that search slopes were steeper during LS (62.1 ± 8.5 ms/item) compared to HS (2.7 ± 2.3 ms/item). We found no significant difference between search slopes from 4-to-8 and 8-to-12 items (p > 0.25). Taken together, these results show that search efficiency was much higher while searching for a high-salient target. In the case of a low-salient target, participants needed an average of 62 ms increase in search time for each added distractor. Whereas, the average increase in search time for an added distractor was close to zero when the target was salient (Table 2).

**Table 2:** Average search times (listed as intercepts) and search slopes for different target saliency conditions and stimulus arousal groups in Experiment 2 (95CI are indicated in parentheses).

Condition	High-Salient Target			Low-Salient Target		
	Intercept [ms]	Slope [ms/item]	Accuracy [%]	Intercept [ms]	Slope [ms/item]	Accuracy [%]
Visual	550 (29)	2.7 (2.3)	98.2 (0.9)	987 (59)	62.1 (8.5)	99.2 (0.5)
Low-arousal	530 (29)	1.8 (2.6)	98.6 (1.2)	979 (72)	63.5 (9.9)	98.2 (1.3)
Mid-arousal	538 (30)	3.3 (2.3)	98.9 (1.1)	966 (65)	58.9 (9.5)	98.6 (0.9)
High-arousal	531 (30)	1.6 (2.7)	98.4 (1.1)	943 (73)	55.1 (10.1)	98.7 (1.1)

The analysis of the accuracy data revealed that participants made fewer mistakes while searching for the low-salient target than for the high-salient target (F(1.25) = 5.06, p = 0.033, η_p_^2^ = 0.17; see Table 2). This finding suggests that fast search times that occur while searching for a high-salient target may come at an accuracy cost. Nevertheless, we did not find this effect in the first experiment or in VA conditions. We found no effect of set size or the interaction between set size and target saliency on accuracy (p > 0.25).

#### Effect of auditory-induced arousal on visual search

To study the effect of the arousal manipulation, we analyzed RTs during VA using an ANOVA with stimulus arousal (low, mid, or high), target saliency, and set size as within-subject factors. As expected, target saliency (F(1.25) = 235, p < 0.001, η_p_^2^ = 0.91) and set size (F(2.50) = 111, p < 0.001, η_p_^2^ = 0.82) main effects and their interaction (F(2.50) = 114, p < 0.001, η_p_^2^ = 0.82) were highly significant. Similar to the analyses of the results in Experiment 1, we looked for significant linear contrasts of the stimulus-arousal factor, in order to investigate the impact of increased arousal on visual attention. As a result, we found significant linear contrasts of the stimulus-arousal main effect (F(1.25) = 4.788, p = 0.038, η_p_^2^ = 0.16) and stimulus-arousal*target-saliency interaction (F(1.25) = 6.236, p = 0.019, η_p_^2^ = 0.2). These results indicated that as auditory-induced arousal increased, the average RTs and the RT differences between the two target-saliency conditions decreased (see intercepts in Table 2). All the other interactions were non-significant. Next, we performed separate analysis for different target saliency conditions. Similar to Experiment 1, linear contrast of the stimulus-arousal main effect was significant for LS (F(1.25) = 5.704, p = 0.025, η_p_^2^ = 0.19). However, this effect did not reach significance for HS (p > 0.25).

Search slopes were analyzed separately for different target saliency conditions using ANOVA with stimulus-arousal (low, mid, or high) and step (slopes between 4-to-8 or 8-to-12 items) as within-subject factors. The linear contrast of the stimulus arousal main effect for LS (F(1.25) = 3.776, p = 0.059, η_p_^2^ = 0.13) indicated that search slopes tended to decrease with increasing stimulus arousal. We found no significant effects for HS (p > 0.25; see search slopes in Table 2). Taken together, these findings indicate that while searching for a low-salient target, increasing arousal induced by the auditory cues caused both intercepts and slopes to decrease.

The analysis of the accuracy data yielded no significant effect of stimulus arousal or interaction between stimulus-arousal and target-saliency (p > 0.25).

#### Valence effects

Similar to Experiment 1, valence and arousal ratings for the auditory stimuli were negatively correlated (Supplemental Table 2). In order to assess whether the results were largely due to stimulus arousal rather than valence, we performed a similar control analysis. First, based on valence ratings we formed high, mid and low-valence stimulus groups for each participant, and computed average RTs (for each stimulus valence, target saliency, and set size) and search slopes (for each stimulus valence and target saliency). Next, we repeated all the ANOVAs listed in the previous section with stimulus valence factor (instead of arousal). As a result, none of the main effects or interactions due to stimulus-valence reached significance (p > 0.1).

## Discussion

The current studies aimed to investigate whether auditory-induced arousal could facilitate visual attention in a search task. In particular, we focused on whether the arousal level induced by an environmental sound as an alerting cue could modulate the auditory-phasic alerting effect on visual attention. In two experiments, we found that, search times decreased as arousal induced by the auditory cues increased while searching for a low-salient visual target. We also found a decrease in search slopes with increasing auditory-induced arousal. Moreover, analyses revealed that speeded responses caused by auditory-induced arousal did not occur at a cost of accuracy. Taken together, current findings present clear behavioral evidence, for the first time, that exposure to affectively arousing environmental sounds can facilitate visual attention and search efficiency in a subsequent visual search. Earlier studies found that exposure to high levels of noise can lead to increased selectivity of attention in central tracking and monitoring tasks (e.g. refs 21 and 22; see ref. 23 for a review) compared to a baseline condition with no auditory stimulus present. These findings point to an alerting effect on visual attention and performance caused by auditory-induced arousal. The findings of the current study on the other hand, reveals clearly the relationship between the level of auditory-induced arousal and attentional facilitation during subsequent visual search; that is, arousal value of the auditory-cue modulates visual attention.

In the first experiment, the main aim was to study whether auditory-phasic alerting could influence search times in a subsequent visual search and whether the level of auditory-induced arousal could modulate this effect. As expected, RTs were significantly lower while searching for a high-salient compared to a low-salient target; and the search times increased as the low-salient target appeared further away from the central fixation. Critical to our hypothesis, RTs decreased with increasing auditory-induced arousal especially while searching for the low-salient target. The findings in Experiment 1 indicate that arousal level induced by the environmental sounds has an impact on subsequent visual search, even when the stimuli are task-irrelevant. We argue that this finding is primarily due to the overall facilitation of attention in the presence of affectively arousing sounds, which in turn has an impact on visual attention. An alternative explanation, however, could be that increased arousal influenced the motor system and caused the response thresholds to decrease. In order to distinguish an effect on response thresholds from an effect on visual search efficiency, we introduced different set sizes in Experiment 2. Therefore, we were able to assess the impact of auditory-induced arousal on search slopes. Moreover, another limitation for the first study is that the findings were based on only four auditory stimuli. Hence, we used 24 different sounds in the second study to investigate whether the effect depends on certain physical features of the four sounds used in the first study or it replicates with other sounds as well. Similar to the first study, search times decreased with increasing arousal induced by the auditory cues, which points to a facilitatory effect of auditory-induced arousal on subsequent visual search. Further, decreasing search slopes with increasing auditory-induced arousal points to an increased search efficiency during the low-salient target condition. Even though we did not find an effect of stimulus arousal on search times for the high-salient target condition (as in Experiment 1), on average participants were slower during VO compared to VA independently of stimulus arousal (see intercepts at Table 2). This might suggest that auditory phasic alerting either influenced the speed of subsequent pre-attentive visual processing, which is modulated first-and-foremost by the visual saliency of the target (ref. 32; see also ref. 13), or it decreased the response thresholds.

It has been suggested that several feature detection mechanisms operate in parallel and encode spatial contrast of each feature to from a two-dimensional salience map of the visual field. Visual attention is used to sequentially scan this salience map^28^. Since spatial contrast between the target and distractors was high during the high-salient target condition, visual attention was guided to the target location almost automatically. Therefore, increasing the number of distractors did not affect the search times in the second experiment. In addition, arousal induced by the auditory cues did not influence the search speed. On the other hand, while searching for the low-salient target, observers needed to deploy attention to limited portions of the visual field because the salience map did not provide a highly probable target location. Unsurprisingly, increased number of distractors caused the search times to increase. However, this process could be modulated by auditory-induced arousal; that is, exposure to more arousing sounds led to faster responses and more efficient search. These results could be understood in the light of the findings that affective stimuli cause a general boost in attention and perception^1^. The presence of affective stimuli could lead to transient changes in attentional thresholds in sensory cortices^2^. Increased arousal can modulate phasic alertness, increase vigilance, and influence neuronal responsiveness of the sensory systems^33, 34^. It has been found that exposure to affective stimuli could both enhance perception and facilitate attention^4^. Furthermore, the presence of arousing stimuli may regulate the activity in the brain regions that are involved in active information processing^1^. Hence, it could be argued that exposure to affective sounds in the current experiments could facilitate visual attention in a subsequent search task by causing a decrease in attentional thresholds. Moreover, exposure to arousing sounds may bias the attentional competition between the target and distractors during the low-salient target condition. In fact, arousal-biased competition (ABC) account proposes that increased arousal would bias the attentional competition in favor of the high priority items^27^. In our case, the high-priority item is the target due to a combination of top-down and bottom-up factors. A very large bottom-up stimulus salience could be the reason for a non-existing effect of sound arousal on search times during the high-salient target condition. On the other hand, in the first study accuracy tended to increase with increasing auditory-induced arousal during high-salient target condition but this effect did not replicate in the second study. Thus, a facilitating effect of auditory-induced arousal on accuracy is not robust.

The auditory system is involved in detection and identification of significant targets in our surroundings, and helping us to orient by guiding the visual system^35^. In this manner, it seems to function like an alarm system^36^. Therefore, exposure to affectively arousing sounds could increase phasic alertness and vigilance, which in turn facilitate subsequent information processing and attention. It has been shown that affective salience of sounds could influence the attentional selection in the auditory domain^7^ and guide the orientation of auditory spatial attention^37^. The novel findings presented here suggest that the attentional effects of auditory-induced arousal could transfer to a visual task as well. In connection to this, recent evidence suggests that auditory attention might have a priming effect on subsequent visual processing^38^.

It is also very important to understand the boundary conditions of this effect. One important issue here might be the timing of auditory and visual stimuli. Since we were interested in the alerting effect of sounds, we presented them before visual stimuli as alerting cues. We also attempted to reduce any effects of perceptual and attentional interference that might be caused by simultaneous presentation of auditory and visual stimuli. Crossmodal interaction studies found higher orientation sensitivity for visual targets when they were presented simultaneously with an auditory stimulus that has high in comparison to low significance^14, 15^. However, those studies argued against an alerting (attentional) effect since brief auditory and visual stimuli were presented simultaneously. Studies on alerting cues use inter-stimulus intervals roughly between 100–900 ms^39^. In our paradigm, simultaneous presentation of auditory and visual stimuli may still lead to attentional benefits, but on the other hand, affective sounds may draw attention and impair task performance. Further experiments are needed to disentangle these possibilities.

Although, we were interested in the alerting effect of auditory stimuli, a small number of previous studies investigated whether the presentation of visual emotional stimuli prior to a visual search could modulate RTs. It has been found that viewing fearful faces prior to performing the search enhanced target detection^5^. However, another recent study using mixed emotionally negative pictures and did not find any effect of arousing stimuli on RTs to detect the visual search target^40^. The current study differs from those studies in a critical aspect; that is, the use of the environmental sounds to induce emotional arousal. We believe the presentation modality could be one of the determining factors for the current findings. Further, when considering effects of arousal on visual search, task difficulty may be another factor to be taken into account. For instance, the low-salient target condition in the current study seems to have a higher visual saliency than the low-salient condition used in the study by Lee and colleagues^40^. This point is also evidenced by the RTs during the low-salient target conditions (in ref. 40 around 1450 ms, while in the current study it is lower than 1150 ms). It has been shown that difficult task conditions (i.e. high perceptual or attentional load) might sometimes override the effects of induced affect and arousal (ref. 41, also see discussion in ref. 42).

A final point that should be made is that even though the aim of the present study was to investigate the impact of auditory-induced arousal on subsequent search, valence and arousal ratings in our data set were correlated. Hence, there is a possibility that stimulus valence, but not arousal, or a combination of the two could be responsible for the findings. We acknowledge that the methods employed in the current study might not be perfectly suited to disentangle these possibilities. However, when we repeated the analyses after ordering the sounds in terms of individual valence ratings, all the reported effects that stem from the auditory-cue factors became non-significant. Hence, stimulus arousal, rather than valence, seems to be responsible for the current findings.

## Methods

### Participants

29 (15 women, 14 men, mean-age = 28.3, SD = 8.83) and 26 (10 women 16 men, age = 28.5 ± 4.86) normal hearing individuals participated in the first and the second experiment, respectively. They gave their informed consent prior to the inclusion in the experiments and were compensated after the studies. The experiments were conducted in accordance with the ethical standards in the Declaration of Helsinki, and were approved by the Västra Götalands regional ethics committee. Participants completed all materials individually in a sound-attenuated room.

### Auditory stimuli

In the first experiment, we used four environmental sounds (Supplemental Table 1), all of which were 2500-ms long. While in the second study, we used 24 different sounds (Supplemental Table 2) that were 2300-ms long. All the four stimuli in the first experiment were used in the second experiment as well. They were edited to have the same duration as the other sounds in the second study (2300 ms). Sounds in both experiments were sampled at 44.1 kHz. Loudness-equalization was done according to fifth-percentile Zwicker-loudness, which was suggested as an index of loudness for temporally-varying, non-impulsive sounds^43^. In the first study, two sounds (growling-dog and fire-alarm) were time-edited versions of the original IADS sounds^44^; and the other two (clucking-hen and microwave-oven) were originally from freesound.org website. For the second experiment, we selected 10 sounds from the IADS database^44^, while the rest were retrieved from freesound.org website. Sounds within each experiment were edited to have the same onset and offset times (All stimuli and other data are available upon request).

Sounds in the first experiment were presented through loudspeaker pairs that were located either in front of or behind participants. Loudspeakers (Genelec8020B) were at 1.2-m height from the ground and at 1.2-m distance from participants. The angle between the loudspeaker pairs were 60° from the participants’ point of view. We introduced a factor of sound source location since a previous study reported an auditory bias towards rear perceptual field at both attentional and emotional levels^31^. Thus, we aimed to investigate a possible source location effect on visual attention. Whereas, in the second experiment sounds were presented only through the front loudspeaker pair.

### Visual search task

In the first experiment, participants actively searched for a visual target among 15 distractors and reported its orientation (left or right) by pressing a respective button on a keypad. The target was a symbol that resembled a letter-T on its either side. Distractors were selected to induce high-salient (HS) and low-salient (LS) target conditions. In HS, the distractors were circles, while in LS they were symbols that resembled letter-Ls in various orientations (Fig. 1A). The orientation of the Ls was unique for each trial in the experiment. All the items were laid out to form a 4 × 4 visual array. During half of the trials, the target appeared in one of the 4 central cells (central-target condition), while in the rest it was in one of the other 12 cells (peripheral-target condition).

In the second experiment, the target and the distractors were the same as in the first experiment. The items were laid out on a circle around the central fixation in order to remove the target location factor (Fig. 3A). Participants actively searched for the target among distractors and indicated its orientation (left or right) by pressing a respective button. Three different set sizes were implemented (4, 8, and 12), and target appearance frequencies were the same for each location within each set size. Visual stimuli in both experiments were displayed on a 20-inch LCD monitor (1280 × 780). The viewing distance was approximately 70 cm, and the display height was adjusted individually to align participants’ eye to the center of the screen. Participants were explicitly instructed to sit straight and not to move away from or towards the display.

### Procedure

In both experiments, participants first listened to the auditory stimuli and were asked to report their momentary affective states (*“How do you feel right now?”*) on visual-analog-scales of valence (positive/negative-affect) and arousal (high/low-arousal) after each sound. Each trial started with a fixation cross (500 ms), which preceded the sound onset. After the sound, participants reported their affective state. In the first experiment, each participant completed one block, in which all the sounds were presented once at each location (front and rear). Two presentations of the same sound were never in succession. Whereas in the second experiment, participants listened to each stimulus once. Participants’ responses in both experiments were scaled between −1 (negative-valence or low-arousal) and +1 (positive-valence or high-arousal).

After the first session, participants in both experiments completed two versions of the visual search task: visual-only version (VO) and the search with auditory cues (VA). The order of the task version was counterbalanced among the participants to control for habituation. In the first experiment, each trial of the VO condition started with a fixation cross (1000 ms), which preceded the visual array (Fig. 1B). Participants actively searched for the target and reported its orientation as quickly and accurately as possible. They were given maximum 3500 ms to find the target. Responses and RTs were recorded. The experimental factors were target-saliency (high or low), target-location (central or peripheral), and target-orientation (left or right). Each of the eight possible conditions was repeated four times, which resulted in a 32-trial block. The other version included the task-irrelevant auditory cues. Each trial started with a fixation cross. The onset of the sound, which was presented through either front or rear loudspeakers, was 500 ms into the fixation period. Thus, the visual array appeared on the screen precisely at the sound offset. The rest of the trial was the same as the visual-only version (Fig. 1C). 8 auditory-conditions (4 sounds, 2 locations) were crossed with 8 search conditions to produce a total of 64. Each condition was repeated twice, which resulted in 128 trials. Participants completed this version in 4 separate blocks to keep the block lengths comparable to the visual-only version.

In the second experiment, each trial in VO started with a fixation period (2500 ms), which preceded the presentation of the search array. Then, participants actively searched for the target and indicated its orientation. Their reaction times (RTs) and responses were recorded. Next trial started after a 3000-ms long ISI (Fig. 3B). The only difference between VO and VA was the presentation of a sound during the fixation period. The sound onset was 200 ms into the fixation period. Hence, the visual array appeared on the screen precisely at the sound offset. For each task version (VO and VA), participants completed three experimental blocks (one for each set size). The block order was balanced among participants. Within each set size, participants went through HS and LS search arrays in random orders (48 trials/block). During the task with auditory cues, each sound was presented once for each set size and target saliency condition.

### Data analyses

In both experiments, average hit-rates during the task with (97.8% and 98.7%) and without the auditory cues (97.4% and 98.6%) were high. The main dependent measure was RT (only correct-trials). However, we also ran analyses on accuracy in order to investigate whether changes in RT come at an accuracy cost. Data analyses provided no significant main effects of or interactions with the target orientation. Thus, RT data was averaged over target orientations and repetitions, after outlier removal. Outliers in the first experiment were defined individually for each combination of target saliency, target location, and the task version as RTs that lie more than two standard deviations outside the mean. 3.1% of data points in VO and 3.2% of data points in VA were removed as outliers. In the second experiment, outliers were defined individually for each combination of set size, target saliency, and task version as RTs that lie more than two standard deviations outside the mean. 4.4% of data points in VO and 4.1% in VA were removed as outliers.

In the first study, we analyzed RTs from the visual-only version of the task using an analysis-of-variance (ANOVA) with target-saliency (HS or LS) and target-location (center or peripheral) as within-subject factors. Then, we focused on the task version with auditory cues. To investigate the influence of auditory-induced arousal on RTs, we first ordered the stimuli based on individual arousal ratings (S1 to S4, see Fig. 2A). Since the most arousing sound might be different for different participants, we ordered the auditory-cues based on the reported arousal-levels. Consequently, S1 is not one particular sound, but instead it is the most arousing stimulus for each participant. Similarly, S4 is the least arousing stimulus for each participant. RT data was analyzed using an ANOVA with auditory-cue (S1-S4), sound-location (front or back), target saliency, and target location factors, which provided the main results critical to our hypothesis regarding the impact of auditory-induced arousal on subsequent visual search. Here, we also looked for *a priori* significant linear contrasts of the auditory-cue main effect and its interactions to study the effect of auditory-induced arousal.

In the second experiment, we first obtained average RTs for each set size and target saliency in VO. From these, individual search slopes were computed for each target saliency condition as a measure of search efficiency. Search slopes were defined as the increase in RT caused by an added distractor to the visual array. We employed a similar analysis strategy in the second experiment to study the effect of auditory-induced arousal on visual search, where we ordered the sounds based on individual arousal ratings. We, then, divided the 24 sounds into three different groups of eight stimuli for each individual (i.e. Low, Mid, and High arousal). Consequently, high, mid, and low-arousal stimulus groups were not composed of exactly the same stimuli for each participant. After forming these groups, we computed mean RTs for each set size, target saliency, and stimulus arousal. Finally, individual search slopes were calculated for each target saliency and stimulus arousal. This analysis strategy was decided prior to data collection and applied to each participant’s data equally. All RTs and search slopes were analyzed using repeated-measures ANOVAs. All reported error terms are 95% confidence intervals unless stated otherwise.

## Electronic supplementary material

Supplementary Information
