# Supplementary material for: Exposure to arousal-inducing sounds facilitates visual search
Source: Sci Rep. 2017 Sep 4;7:10363. doi: 10.1038/s41598-017-09975-8 (PMC5583323; doi:10.1038/s41598-017-09975-8)
Supplement: Supplementary file 1 — Supplementary Information [file 41598_2017_9975_MOESM1_ESM.pdf]

Exposure to arousal-inducing sounds facilitates visual search

Erkin Asutay [1, *], Daniel Västfjäll [1, 2]

1. Department of Behavioral Sciences and Learning, Linköping University, Linköping, SE-58183, Sweden.

2. Decision Research, Eugene, OR, 97401, USA

* Corresponding author. Tel. +4613281844, email: erkin.asutay@liu.se

Supplemental Information

Supplemental Table 1. Affective reactions to auditory stimuli in Experiment 1.

**Stimulus**	**Mean valence (95% CI)**		**Mean arousal (95% CI)**	
	**Front**	**Back**	**Front**	**Back**
Growling dog	-.31 (.13)	-.25 (.14)	.33 (.14)	.33 (.15)
Fire alarm	-.47 (.11)	-.43 (.12)	.45 (.14)	.46 (.14)
Clucking hen	.17 (.17)	.12 (.16)	.06 (.18)	-.04 (.17)
Microwave oven	-.06 (.14)	.07 (.13)	.01 (.13)	-.05 (.16)

Note: Valence and arousal ratings are scaled between -1 and +1. CI=confidence interval.

Supplemental Table 2. Mean valence and arousal ratings for each auditory stimulus, together with the number of times of each sound classified in respective stimulus arousal categories. Valence and arousal ratings are scaled between -1 (negative-valence or low-arousal) and +1 (positive-valence or high-arousal). The numbers in parentheses represent standard error of the means. The correlation between valence and arousal ratings was assessed after pooling the data over participants and stimuli. Valence and arousal ratings were negatively correlated (r=-0.45, p<.001, N=624).

**Stimulus Description**	**Valence**	**Arousal**	**Low arousal count**	**Mid arousal count**	**High arousal count**
Boiling water	-0.03 (.08)	-0.02 (.07)	15	7	4
Buzzer	-0.38 (.08)	0.41 (.08)	1	6	19
Clucking hen	0.16 (.07)	0.01 (.07)	12	10	4
Compressed air	-0.33 (.08)	0.22 (.07)	7	9	10
Cracking door	-0.27 (.06)	0.13 (.09)	10	9	7
Crushing a tincan	-0.02 (.06)	0.14 (.05)	6	15	5
Crying baby	-0.42 (.08)	0.35 (.08)	2	9	15
Cuckoo clock	0.03 (.07)	0.24 (.05)	3	12	11
Dentist drill	-0.53 (.07)	0.33 (.06)	3	12	11
Female scream	-0.63 (.09)	0.43 (.09)	3	3	20
Female yawn	0.09 (.06)	-0.33 (.09)	19	3	4
Fingernails on blackboard	-0.49 (.07)	0.27 (.06)	4	12	10
Fire alarm	-0.26 (.08)	0.43 (.09)	4	4	18
Growling dog	-0.27 (.07)	0.27 (.09)	4	8	14
Helicopter	-0.01 (.08)	0.00 (.08)	14	9	3
Hissing cougar	-0.26 (.07)	0.23 (.07)	5	11	10
Horse hooves	0.33 (.06)	0.05 (.06)	13	7	6
Jackhammer	-0.33 (.07)	0.07 (.07)	12	7	7
Microwave oven	0.00 (.09)	-0.16 (.07)	21	5	0
Roaring tiger	-0.06 (.07)	0.21 (.09)	6	9	11
Scratching styrofoam	-0.43 (.06)	0.17 (.07)	5	18	3
Toilet flush	-0.15 (.06)	-0.17 (.07)	17	8	1
Typrewriter	0.01 (.06)	0.07 (.07)	12	7	7
Wasps	-0.23 (.09)	0.10 (.09)	10	8	8
